# Approximating the maximum tibial coverage in total knee arthroplasty does not necessarily result in implant malrotation

**DOI:** 10.1038/s41598-020-67613-2

**Published:** 2020-06-29

**Authors:** Long Shao, Xiang-Dong Wu, Ting Wang, Xiao-Kang Liu, Wei Xu, Wei Huang, Zhi-Min Zeng

**Affiliations:** 1 Department of Orthopaedic Surgery, Ningbo No. 6 Hospital, 1059 Zhongshan East Road, Ningbo, Zhejiang China; 2 Department of Orthopaedic Surgery, Peking Union Medical College Hospital, Chinese Academy of Medical Sciences and Peking Union Medical College, Beijing, China; 3 Department of Orthopaedic Surgery, The First Affiliated Hospital of Chongqing Medical University, No. 1 Youyi Road, Yuan Jiagang, Yuzhong District, Chongqing, China; 4 Lab of Computer Vision, School of Electronics and Information Communications, Huazhong University of Science and Technology, 1037 Luoyu Road, Wuhan, China

**Keywords:** Bone, Orthopaedics, Medical research

## Abstract

Traditionally, the practice of the tibial component placement in total knee arthroplasty has focused on achieving maximum coverage without malrotation. However, the concept of maximizing coverage has not been well defined or researched and yet biased results are often produced. This study aimed to evaluate the effect of a prioritizing maximum coverage positioning strategy on the rotational alignment by using a strict computer algorithm. Computed tomographic scans of 103 tibial specimens were used to reconstruct three-dimensional tibia models. A virtual surgery was performed to generate the resection plane with a posterior slope of 7° on the proximal tibia. Symmetrical and anatomical tibial components were placed and analyzed with an automated program designed for approximating the maximum coverage based on the coherent point drift algorithm. We found that the average tibial coverage achieved across all specimens and implants was 85.62 ± 3.65%, ranging from 83.64 ± 4.10% to 86.69 ± 3.07%. When placed for maximal tibial coverage, the mean degree of rotation related to the Insall line was − 0.73° ± 4.53° for all subjects, 23% of the tibial components were malrotated. The average percentage position of the baseplate anteroposterior axis over the patellar tendon was 26.95 ± 14.71% from the medial edge. These results suggest that with specific design and proper placement of the component, approximating the maximum tibial coverage in total knee arthroplasty does not necessarily result in implant malrotation. The current tibial baseplates have shown good performance on the coverage when aligned parallel to the Insall line with the anteroposterior axis positioned between the medial 1/3 and medial 1/6 of the patella tendon.

## Introduction

Insufficient implant coverage and bone support at the osteotomy level could increase the amount of bleeding into the articular cavity of the knee in the immediate postoperative period, which may also increase osteolysis from wear by debris in the long term follow-up, finally resulting in subsidence and instability^1,2^. Meanwhile, proper tibial rotation is another key factor that is crucial to the kinematic alignment and long-term survivorship of the prosthesis^3–5^. Ideally, a satisfying total knee arthroplasty (TKA) ought to maximize tibial coverage without causing tibial malrotation.

Several studies have evaluated both tibial coverage and rotational alignment with virtual matching by using preoperative imageological data^6–13^. These studies mainly focused on the comparison between anatomic and symmetric designs which had always been a controversial issue. However, the various definitions of the malrotation and coverage maximizing methods applied in these studies make it hard to draw a firm conclusion on the compromise between coverage and ideal rotation while the latter has an inherent priority^8,14^. Theoretically, maximizing coverage usually requires a larger size of the tibial baseplate, which under normal circumstances would most likely result in overhang and corresponding repositioning in term of rotation. Thus, the surgeon’s task of choosing the optimum tibial baseplate size and alignment can be compromised when confronting the incongruence between coverage and alignment, as illustrated by Bonnin et al.^15^. Most orthopedic researchers have generally recognized that it is impractical to simultaneously achieve maximum coverage without implant malrotation or overhang in TKA^6,8–10,14^. To overcome this tradeoff, prosthesis with a rotating platform was advocated by some authors, which allows tibial coverage to be maximized without adversely affecting correct alignment^16,17^. However, when using conventional prosthesis in a standard TKA procedure, many joint surgeons are still wondering which orientation should the tibial baseplate be set and whether there is a position of the orientation axis over the tibial tubercle that can be easily located. Also, the question of how to strike a balance between maximizing tibial coverage and proper rotation remains unsolved.

Therefore, by using a strict computer algorithm, the purpose of this study is (1) to evaluate the degree of tibial baseplate rotation when placed to maximize coverage and (2) to also evaluate the location of the baseplate anteroposterior (AP) axis over the patellar tendon. Our primary hypothesis was that approximating maximum tibial coverage at the bone-implant contact interface level would be accomplished without malrotation of the implant.

## Methods

### Patients

This prospective study was observational and non-therapeutic, it was approved by the Ethical Committee of First Affiliated Hospital of Chongqing Medical University and the Ethical Committee of Ningbo No.6 Hospital. The methods were carried out in accordance with the relevant guidelines and regulations, and informed consent was obtained from all participants. In this study, 56 volunteers were enrolled consecutively from April 2016 to August 2019. Both knees of each subject were studied. Inclusion criteria comprise healthy knees without any symptoms of soft tissue injuries or osteoarthritis. This was verified both via clinical examination and examination of computer tomographic (CT) images. Exclusion criteria consisted of varus or valgus knees, arthritic changes, bone defects, prior trauma or surgical history, and congenital deformities. At last, four subjects were excluded because of abnormal tibial tubercle position. One knee from another subject was found to have a bone defect and excluded. A total of 103 knees from 52 volunteers, including 26 males and 26 females, were analyzed in this study. The age of the volunteers ranged from 20–66 years (mean 42.96 ± 17.23 years).

### Virtual osteotomy on a modified coordinate system

A CT scan of the knee was performed to acquire 1.0 mm CT slices (resolution, 512 × 512 pixels) from the femur head to the center of the ankle joint on a helical CT scanner (Somatom Definition Flash; Siemens Healthineers, Siemens, Germany) according to a standard protocol. The subject was supine with his or her knee in a relaxed and extended position during the scan. A 3D bone model of the lower extremity was reconstructed from original Digital Imaging and Communications in Medicine (DICOM) data using the specialized image processing software Mimics 17.0 (Materialise, Belgium).

**Figure 1:**
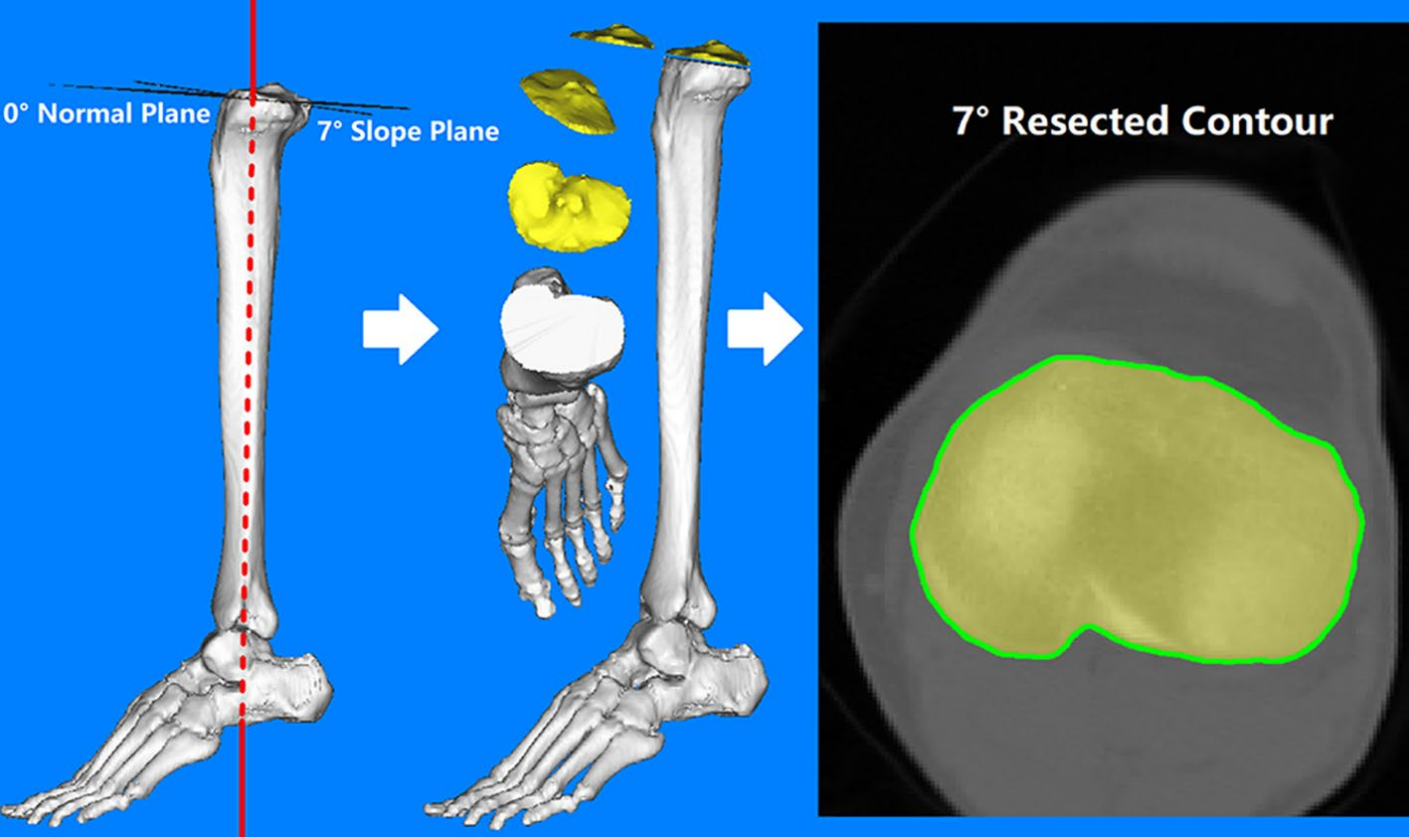
A simplified flow diagram of the virtual osteotomy and the resected contour extraction from a three-dimensional bone model. Red line: the longitudinal axis of the tibial shaft which was used to calculate the 0° normal plane.

**Figure 2:**
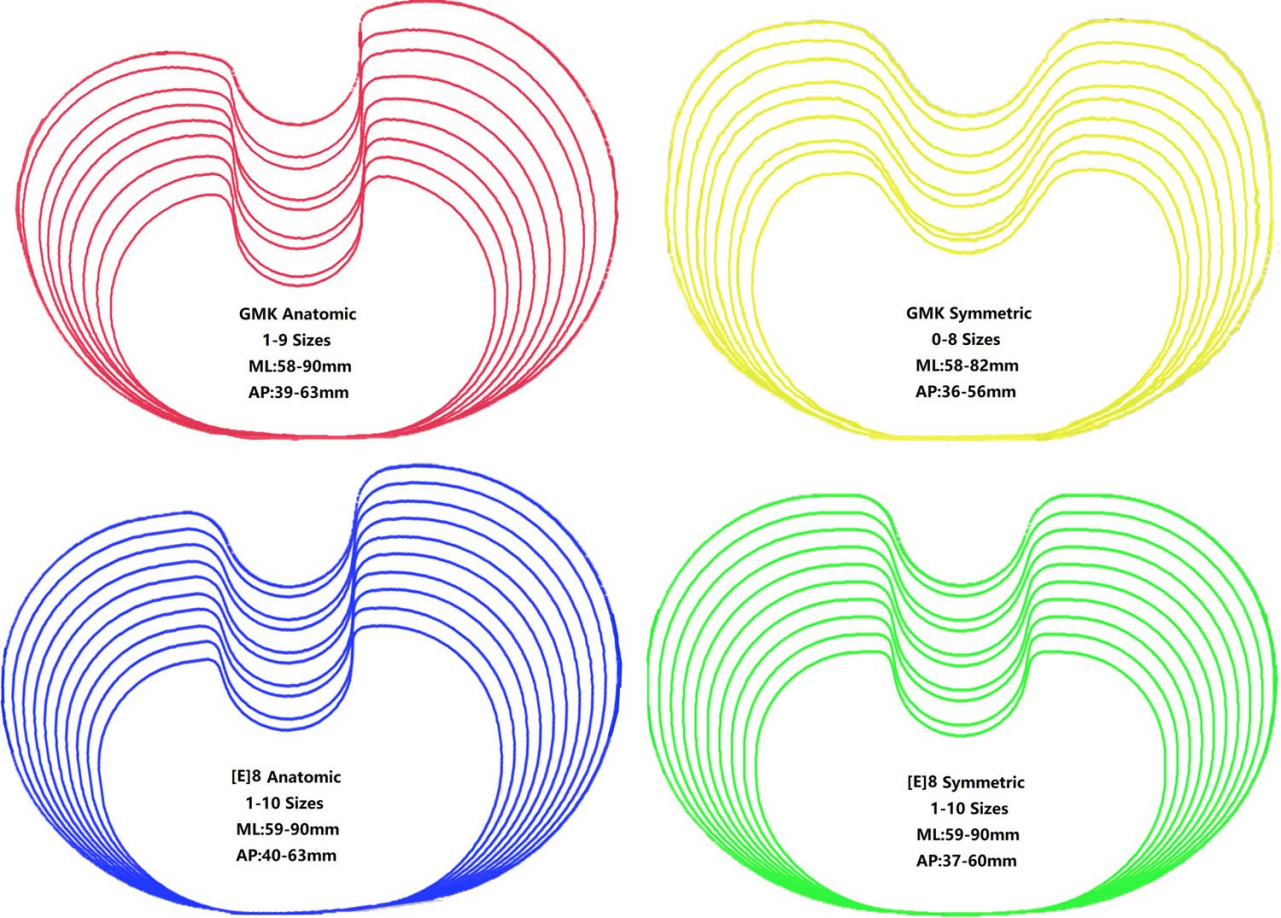
The four tibial baseplate designs, their associated sizing distribution and geometric measurements.

A coordinate system was modified by referencing the method of Kuwano^18^ and Ma^19^. In this modified coordinate system, each of the 3D tibia specimens was repositioned with a standard translation and rotation procedure before the virtual osteotomy. A virtual surgery was then performed on the proximal tibia at a level 8 mm below the lateral tibial plateau with a posterior slope of 7°, which had been found to be a superior slope degree for tibial component placement based on a previous study^20^ (Fig. 1). All the coordinates and angles were generated, calculated, and examined using space vector and mathematics for precision. Along with the width of the patellar tendon and the posterior cruciate ligament (PCL) insertion site, the outer cortex of the tibial bone at the resection plane were all extracted to create a full resection contour for each subject. Four tibial baseplate designs, including symmetrical and anatomical types (Link Gemini Mobile Knee series and Link [E]8 series, LINK, Germany) with different design attributes, were prepared for assessment. Baseplate contours were also extracted from all the available sizes, and the overall mediolateral and anteroposterior dimensions of the tibial baseplate were measured (Fig. 2). The main differences across these designs were that the anatomic design had a larger medial tibial plateau, and the shape of the PCL zone was different. All the contours were extracted together with a sizing marker to prevent magnification error.

### Contour registration based on CPD algorithm and validation

An automated program was developed in Matlab™ (Mathworks, Natick, MA, USA) and used to optimize the registration of the tibial baseplate contours and the resected plateau contours by minimizing the amount of overhang and undersize area in each matchup. In our research, the largest size of the baseplate was chosen as the program automatically calculate the distance between two contours along the standard anteroposterior and mediolateral vector with a maximum tolerance of 2 mm cortical overhang^6,10^. Then, the principal axes of the baseplate contour and the tibial resection contour were extracted separately and positioned in parallel with each other using the principal component analysis in a least-squares sense. This step was considered a rough matching in order to provide starting centroid coordinates for contour registration. A further rigid transformation of the initial matchup using the coherent point drift (CPD) algorithm based on shape registration was carried out in the program, including automatic adjustments for both translation and rotation to simulate the surgical practice of maximizing tibial coverage as may be done in a TKA. Approximating maximum coverage in the present study was achieved theoretically because (1) the largest size of baseplate was used as the number of sizes was limited; (2) the contours maximal matching was completed by the rigorous CPD algorithm which was objective and stable; (3) there is no possible subquadratic time algorithm for the absolute maximum overlap of general polygons as it is a 3SUM-Hard problem in computational complexity theory^21,22^. Along with the automatic matching process, one senior surgeon with extensive experience in TKA independently determined the placement of each tibial baseplate contour on the corresponding resection contours. The rotation difference between the baseplate AP axis and the Insall line (a widely used intraoperative aligning reference that connects the medial 1/3 of the patellar tendon and the projected PCL insertion site) was measured and recorded for further validation of the algorithm with a sample size of 140 (k = 2, power = 90%, alpha = 0.05).

### Coverage, PCL zone, rotation, and percentage position

**Figure 3:**
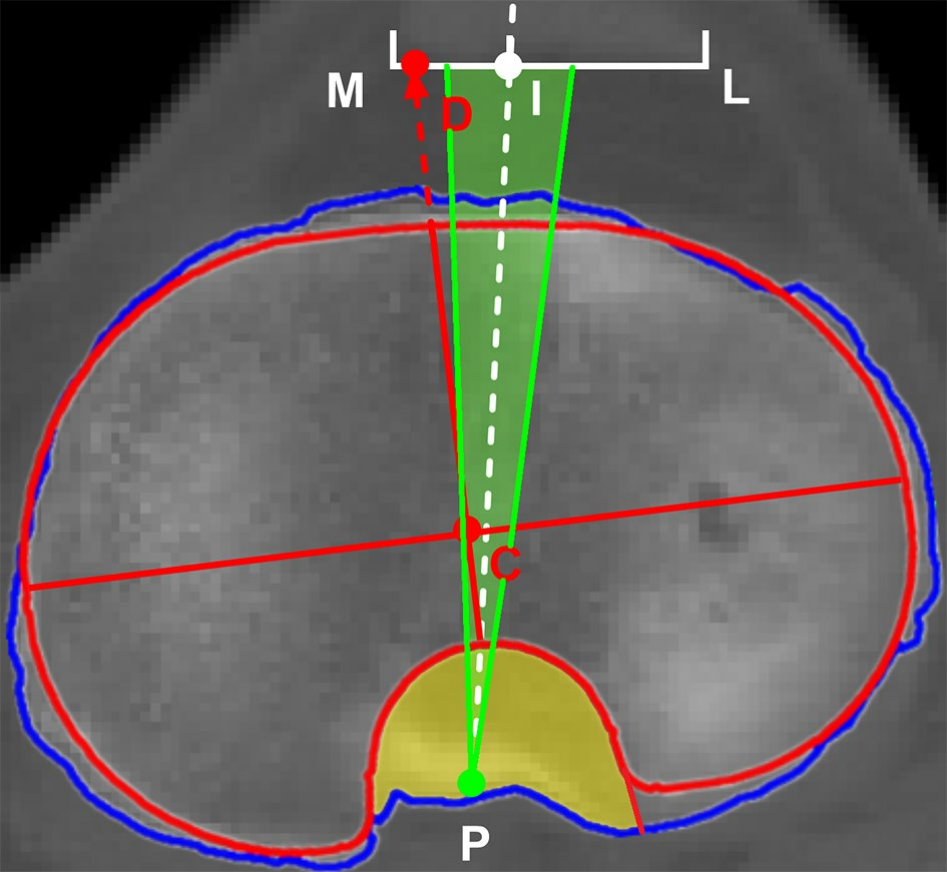
The composite image of the matched contours over the corresponding resected plane. The line CD was defined as the AP axis of the baseplate. The line PI was defined as the Insall line. The angles between the AP axis and the line PI were measured and recorded as the rotational degree. The percentage position of the baseplate AP axis over the patellar tendon was defined as the length of MD divided by the length of ML. The yellow area indicated the uncovered posterior cruciate ligament (PCL) zone. The green area indicated ± 5° rotation interval with respect to the Insall line. ML: the patellar tendon width. C: The centroid of the baseplate. D: The intersecting point of the baseplate AP axis extension over the patellar tendon. I: The medial 1/3 point of the patellar tendon. P: The projected middle of the PCL insertion site.

Together with another senior arthroplasty surgeon, two surgeons checked on the matchup result to make sure that the mismatch, including overhang and undersize, were clinically acceptable. Then the reconfirmed composite image of the matched contours was loaded back to the program again for further measurement of the interface coverage, rotation degree, and percentage position of the baseplate AP axis over the patellar tendon, respectively. The coverage was defined as the total cross-sectional area (CSA) of baseplate minus any overhang, divided by the total CSA of the corresponding tibial surface. The uncovered PCL zone was also calculated as the CSA of the zone divided by the CSA of the tibial surface. The rotation degree was defined as the angle between the baseplate AP axis and the Insall line, in which a positive value represented the lateral rotation. When the AP axis of the implant was not aligning within ± 5° of deviation from the Insall line, the malrotation was considered, referencing the studies by Martin et al.^8^ and Stulberg et al.^9^. The distance between the medial edge of the patellar tendon and the intersection point where the implant AP axis going across the patellar tendon was measured. Divided by the full length of the patellar tendon, a percentage position of the intersection point was recorded as the positive value representing a lateral position (Fig. 3).

### Statistical analysis

Data analysis was performed by an independent statistician using SPSS (Version 22; SPSS Inc, Chicago, IL, USA). The quantitative data is expressed as the mean ± standard deviation (x ± s), and the qualitative data is expressed as a percentage. Analysis of the matchup data was performed by using a randomized block design analysis of variance (ANOVA). If the ANOVA test revealed a statistically significant difference among groups, a comparison of each matchup was performed using the paired t-tests. The qualitative data were compared using the Chi-square test. The Shapiro–Wilk test was used for the determination of the normal distribution of the data. The Intraclass correlation coefficient (ICC) was applied to measure the extent of agreement and consistency. The level of significance was defined at p-value < 0.05.

## Results

The algorithm validation test showed that the Cronbach’s Alpha and ICC for consistency between the human and computer simulation on the rotational degree of the prosthesis for each contour registration was 0.926 and 0.861 (95% CI 0.812–0.899, F = 13.429, p < 0.001).

**Figure 4:**
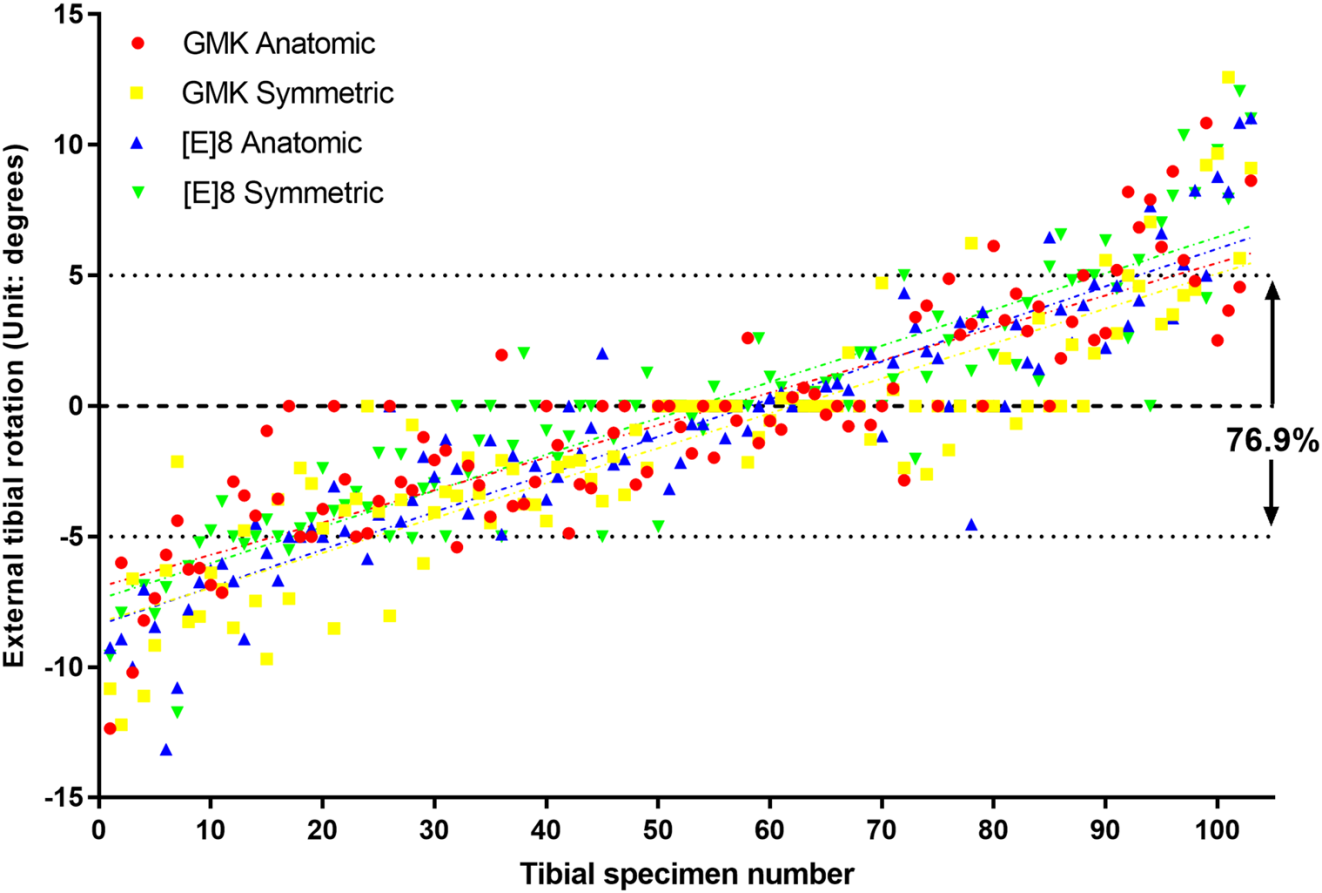
The degree of rotation for each specimen according to tibial component design when the Insall line was set as the reference axis with tibial rotation of 0°. The majority (76.9%) of the specimens were properly rotated within the ± 5° interval.

The angle between the medial aspect of the patellar tendon and the Insall line was 8.58° ± 1.10°. The mean degree of rotation related to the Insall line was − 0.73° ± 4.53° for all subjects. However, the GMK symmetric design along with the [E]8 anatomic design, showed a more internally rotated alignment compared with the other designs, as shown in Table 1 (p < 0.01). When placed for maximal tibial coverage, only 23% of the tibial components were malrotated (Fig. 4), and the actual numbers of malrotated subjects for each design were similar (Table 1).

**Table 1:** The mean ± SD of percent coverage, uncovered PCL zone, degree of rotation and percent malrotation tabulated according to implant design.

Design	Coverage (%)	Uncovered PCL zone (%)	Degree of rotation related to the Insall line (range)	Percent malrotation (number of the malrotation subjects)
GMKA	83.64 ± 4.10	8.08 ± 2.02	− 0.49 ± 4.25 (− 12.35 to 10.84)	20.39% (21)
GMKS	85.54 ± 3.13	7.25 ± 1.85	− 1.35 ± 4.57 (− 12.21 to 12.60)	24.27% (25)
[E]8A	86.60 ± 3.40	6.92 ± 1.95	− 0.89 ± 4.69 (− 13.15 to 11.03)	24.27% (25)
[E]8S	86.69 ± 3.07	7.08 ± 1.76	− 0.18 ± 4.58 (− 11.74 to 12.06)	23.30% (24)
Overall	85.62 ± 3.65	7.33 ± 1.95	− 0.73 ± 4.53 (− 13.15 to 12.60)	23.06% (95)

The positive value represented the external rotation referring to the degree of rotation.

p < 0.001 GMKA versus GMKS, [E]8A, [E]8S coverage; GMKA versus GMKS, [E]8A, [E]8S PCL Zone; GMKS versus [E]8S degree of rotation.

p < 0.01 GMKS versus [E]8A, [E]8S coverage; GMKA versus GMKS degree of rotation; [E]8A versus [E]8S degree of rotation.

p < 0.05 GMKS versus [E]8A PCL Zone.

*GMKA* the Gemini Mobile Knee anatomic design, *GMKS* the Gemini Mobile Knee symmetric design, *[E]8A* the [E]8 anatomic design, *[E]8S* the [E]8 symmetric design.

The average tibial coverage rate, in the maximizing situation, achieved across all specimens and implants was 85.62% ± 3.65%, ranging from 83.64% ± 4.10% to 86.69% ± 3.07% (Table 1). The Gemini Mobile Knee (GMK) system showed a worse performance on coverage compared to the [E]8 system (p < 0.01). Meanwhile, the GMK series showed a more uncovered PCL zone after virtual matching with the tibial components, especially for the anatomic design (p < 0.001), as shown in Table 1.

**Figure. 5:**
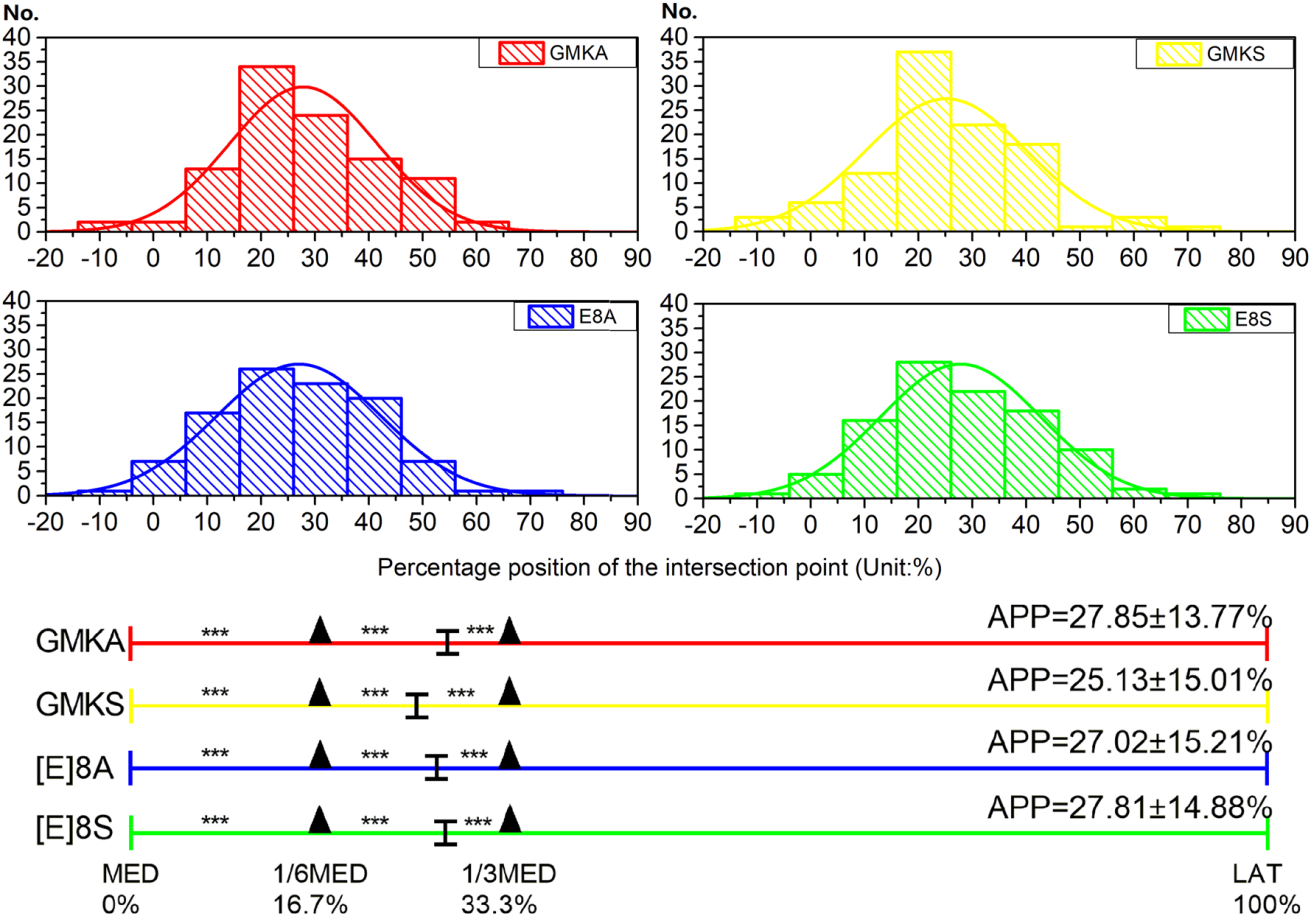
Distribution of the percentage position of the baseplate AP axis over the patellar tendon. The average percentage position (APP) was 26.95 ± 14.71% of the full patellar tendon from medial edge. Curve line in colors indicated the normal distribution. The positive value represented the lateral position. *No.* the count number of the subjects, *GMKA* the Gemini Mobile Knee anatomic design, *GMKS* the Gemini Mobile Knee symmetric design, *[E]8A* the [E]8 anatomic design, *[E]8S* the [E]8 symmetric design. *MED* medial edge of the patellar tendon, *1/6MED* medial 1/6 of the patellar tendon, *1/3MED* medial 1/3 of the patellar tendon, *LAT* lateral edge of the patellar tendon. ***p < 0.001 (Paired T test).

The average percentage position of the implant AP axis was 26.95% ± 14.71% of the full patellar tendon from the medial edge. Although the Shapiro–Wilk test showed that the data for each design had a normal distribution respectively, the variability was extensive, ranging from − 13.43 to 72.76%, as shown in Fig. 5. It also illustrated the percentile results that the maximizing coverage positions for different designs were all located between axis medial 1/3 and axis medial 1/6 of the patella tendon. There were differences between all the adjacent axes with statistical significance, as shown in the Fig. 5 (p < 0.001).

## Discussion

The minimal coverage rate reported in previously published studies was 75–85%^23,24^. However, to maximize the tibial coverage, the choice of baseplate from all available sizes in any commercial artificial knee systems should be as large as possible. Because an undersized baseplate will surely fit the tibial cut surface without any overhang but adversely induce insufficient interface coverage and potential malrotation. On the other hand, it is still unclear to what extent of tibial overhang will lead to clinical symptoms requiring revision surgery, some studies have found that sizing of the tibial components does not significantly affect postoperative outcomes, less than 3 mm of overhang were considered mild overhang and can be acceptable in some cases^2,6,10,25–28^. Based on the above premises, both manual and automatic positioning for tibial baseplate has been reported previously. Martin et al. invited three observers to direct the sizing and positioning of the implant and found out that maximizing tibial coverage would result in implant malrotation^8^. However, the concept of optimal and maximum coverage was confusing, and the conclusion was not convincing if the positioning was simply based on visual observation. Some researchers used commercial software to determine the position of the baseplate without a full understanding of the rationale, and most of the details related to the positioning process were missing^9,13^.

A well-designed algorithm may be able to solve the problem more efficiently and scientifically. Actually, the abstraction of the contours maximum matching problem had been proved to be unsolvable, especially with general polygons like the outer profiles presented in most related studies, including ours^21,22^. Therefore, the idea of “maximum” or “maximizing” described in previous publications with solely medical background could, to some extent, be misleading^6,8,9,13^.We believe approximating the maximum is more appropriate when describing the problem. For various reasons, studies that clearly described the algorithm for achieving the “maximum” coverage were rare. An algorithm introduced by Clary et al. was the only one we could find in which they placed points uniformly around the periphery of the tibial base and measured the distance between these coupled points^6^. Theoretically, what they did was using the Iterative Closest Point algorithm to minimize the sum of square distances between the coupled point sets in order to get their “maximum” matchup. However, compared with the limited boundaries distance calculation, the consideration of a general shape resemblance is a more appropriate way since the latter will not significantly be affected by the initial marking point distribution and noise during the process. First presented by Myronenko and Song^29^, the CPD algorithm has been proved to be one of the most efficient algorithms for point set registration^30^. It considers the alignment of two point sets as an estimation of probability density function and registers point sets by fitting Gaussian mixture models to their centroids and maximizing their likelihood. Thus, the up-to-date algorithm had been used in the fields of medical research, such as bone and vessel three-dimensional model registration and shape matching^31,32^. The maximum coverage was approximated by using the CPD algorithm in our study, and the ICC analysis showed an excellent level of agreement between human and machine measurements, indicating that the algorithm was reliable and stable comparing to manual matching.

Placing the tibial component intraoperatively with both maximum surface coverage and proper alignment is never easy due to the prosthesis deficiency, positioning strategies, and individual variances. As has been noted, Martin et al. reported a level of as much as 70% of all tibial components placed in internal malrotation (average 9°) with maximizing fit^8^, for two symmetric designs, the malrotation rate was almost 100%. Clary et al. demonstrated that maximizing positioning would induce variability in tibial base alignment and should be avoided even though they did not define the malrotation in specific^6^. The situation was quite different in our study that the malrotation percentage went down to only 23% instead. Also, Stulberg et al.^9^ reported a minimum of 73% of cases within proper rotation using the same Insall line reference as ours. Hirakawa et al. found that the tibial component was aligned within the medial one-third of the patellar tendon in 77.7% knees^13^. These were consistent with our results implying that approximating maximum tibial coverage does not necessarily result in implant malrotation. The improvement in the malrotation rate may be attributed to the specific design modification, increased number of baseplate sizes, and proper positioning strategies. Nevertheless, the anatomic difference between healthy individuals in our study and patients with degenerative changes from previous researches should also be noted since it might have an impact on the level of resection and the surgical approach, which may further influence the rotational alignment of the tibial prosthesis^33,34^. It is also worth noticing that original researches related to aligning reference, prosthesis design, and surgical technics in the modern TKA system was usually based on normal anthropometric measurements^7,35^, that is why we enrolled healthy subjects in order to provide a perspective on malrotation from normal conditions.

Our results showed an average 0.73° internal rotation related to the Insall line for all implants placed with maximum coverage. Similar rotational interval was also confirmed by Stulberg et al.^9^, who performed virtual studies using different TKA knee systems from ours. Barrack et al. found averaging 6.2° of internal rotation to be associated with anterior knee pain^36^. Meanwhile, Ushio et al. indicated that the AP axis of the proximal tibia might be significantly internally rotated after proximal tibial resection^37^. The angle between the medial aspect of the patellar tendon and the Insall line was 8.58° on average in our research. To avoid internal malrotation, it seems the best AP orientation when placing the tibial component should be parallel to the Insall line for our subjects, a more lateral alignment, rather than the Akagi line (connecting the PCL insertion site and the medial edge of the patellar tendon) which was said to be more compatible with Asian patients^38^. What is more, the percentage position of all implant AP axis over the patellar tendon was 26.95% on average, which is close to the intersection point of the Insall line. Ma et al.^19^ also found a similar position with a different anatomical prosthesis, which strongly supported our findings. The percentage position analyzed in our study was different from the baseplate orientation that it could help direct the mediolateral translation when a specific rotational alignment has already been set in clinical practice. A high level of the consistency between axial rotation and percentage position would help ensure the prosthesis efficiency across the patient population.

Differences in coverage were statistically compared and found significant across different prosthesis designs in our research (Table 1). The performance of the [E]8 series in coverage was significantly improved when comparing to the GMK series. The GMK anatomic design had the lowest coverage comparing to the other designs, mainly because of the most significant 8% uncovered PCL zone it presented (p < 0.001). Interestingly, approximately 7% size of the tibia plateau would be enough for the PCL insertion based on previous researches, which is consistent with our findings^39,40^. Since the PCL zone had a significant impact on the coverage by definition, we recommend that the size of the posterior notch area should be strictly limited when designing the tibial baseplate profile. Sincerely, the difference between symmetric and anatomic designs on coverage and malrotation rate in our research was not significant. However, numerous studies have reported conflicting results on the choice between symmetric and anatomic designed TKA systems^7–11,41^, the controversial issue had been and would be non-stop as long as every researcher stick to their position and preference. One of the critical differences between symmetric and anatomic designs was that the anatomic design usually had a larger medial tibial plateau simulating the normal knee anatomy. What we found with our resected plane profiles, cases like the same size tibial plateau or even smaller medial plateau did exist. That is to say, the overhang would most probably happen in the medial plateau when anatomically designed baseplate was improperly applied in those cases. Thus, we suggest that surgeons should be aware of this possibility and remain neutral to the prosthesis choice.

The current study is unique in that the matching algorithm was reliable and objective to produce solid results. Also, it is a useful exploration into the application of computational algorithms on the medical problem. Nevertheless, it still presented some additional limitations. First, our volunteer cohort was healthy and relatively young, and patients with degenerative changes might lead to a different result. Second, the program we designed was not capable of identifying certain osteophytes or bone defects that may influence the matching process or placement of the baseplate. Further studies are needed to explore what modifications in prosthesis design could improve its morphologic fit to the tibial resection plane and whether it is possible for the surgeon’s practice to carry out the algorithmic planning fully.

## Conclusion

With specific design and proper placement of the component, approximating the maximum tibial coverage in a TKA does not necessarily result in implant malrotation. The LINK tibial baseplates used in our study, regardless of prosthesis design, have shown good performance on the coverage with an average of 85.62% when aligned parallel to the Insall line and the extension cord of the AP axis positioned between the medial 1/3 and medial 1/6 of the patella tendon.

## Data Availability

The datasets generated and analyzed during the current study are available from the corresponding author on reasonable request.
